# *Plasmodium* telomere maintenance: uncovering the Achilles’ heel for novel antimalarials

**DOI:** 10.3389/fcimb.2025.1659175

**Published:** 2025-09-10

**Authors:** Theophilus N. Wakai, Dorathy O. Anzaku, Israel S. Afolabi

**Affiliations:** ^1^ Department of Biochemistry, College of Science and Technology, Covenant University, Ota, Nigeria; ^2^ Covenant Applied Informatics and Communication Africa Centre of Excellence (CApIC-ACE), Covenant University, Ota, Ogun State, Nigeria

**Keywords:** malaria, telomeres, plasmodium, telomerase, drug resistance, therapeutic target

## Abstract

This review examines the potential of disrupting telomere maintenance in *Plasmodium* as a novel antimalarial strategy. Telomeres are repetitive DNA–protein structures located at chromosome termini, where they preserve genome stability and protect against degradation. Telomere maintenance is crucial for rapid growth, genome integrity, and immune evasion in *Plasmodium* parasites. Unlike humans, *Plasmodium* maintains continuous telomerase activity and uses unique telomere-binding proteins across its lifecycle. These features drive parasite virulence and antigenic variation. Emerging evidence suggests that *Plasmodium* telomeres harbor G-quadruplex (G4) DNA structures, which help stabilize telomeres during replication and may be good targets for small molecules to disrupt their function. Additionally, the parasite depends heavily on its telomerase catalytic subunit, PfTERT, for survival. Inhibiting PfTERT has shown promising results in blocking telomere elongation and impairing replication. Targeting this parasite-specific telomere–telomerase axis may offer a strategic means to destabilize chromosomes, weaken immune evasion, and limit parasite survival, making it a promising antimalarial approach. However, researchers must consider the risks of off-target effects in future drug designs. Though current studies are limited and remain inconclusive, we suggest that future research should investigate combining telomere-directed therapies with existing antimalarials to help overcome resistance and improve treatment outcomes. Herein, we review advances in understanding *Plasmodium* telomere biology, highlighting its distinct structures, critical telomere-associated proteins, and roles in pathogenesis. We further explore how selective targeting could exploit an Achilles’ heel in parasite survival, offering fresh possibilities for next-generation, parasite-specific malaria therapies.

## 1 Introduction

Malaria remains a global health crisis, with 263 million cases and 597,000 deaths in 2023, predominantly in the WHO African Region (World Health Organization, 2023). Children under five account for ~80% of fatalities in this region. Rising drug resistance in *Plasmodium* species threatens existing treatments, necessitating novel therapeutic targets (Ashley et al., 2014; Menard and Dondorp, 2017) This highlights the urgent need for effective interventions to address and mitigate the devastating impact of malaria, especially on the most vulnerable populations. This disease hampers the staggering economic potential of Africa and parts of Asia, and continual exposure selects for a global gene pool of the parasite that develops resistance to first- and second-line chemotherapy (Sinha et al., 2014; Pimenta et al., 2022).

Despite significant advances in the development of anti-malarial drugs and vector control strategies, the fight against malaria is far from over. The continuous emergence of drug-resistant strains of *Plasmodium* poses a significant challenge to existing treatment regimens (Ashley et al., 2014; Ippolito et al., 2021). High transmission rates contribute to the spread and re-emergence of malaria in areas where it was once eradicated, particularly in high-endemic regions (Adegbite et al., 2021; Olasehinde et al., 2015; Oranusi et al., 2025). In addition, the rise in drug resistance in *Plasmodium* species threatens existing treatments, necessitating a search for novel therapeutic targets (Ashley et al., 2014; Menard and Dondorp, 2017; Adegboro and Afolabi, 2024). Amidst this search for molecular targets, one area gaining increased attention is the parasite’s genome maintenance machinery, particularly the role of telomeres in sustaining *Plasmodium*’s survival and virulence (Religa et al., 2014) (Bachmann et al., 2019).

While many strategies have focused on metabolic enzymes, transporters, or oxidative stress pathways (Kumar et al., 2018; Shibeshi et al., 2020; Buthelezi et al., 2025) telomere biology represents a fundamentally different type of vulnerability. In *Plasmodium falciparum*, telomeres not only safeguard chromosome ends but also play a direct role in regulating subtelomeric virulence genes such as *var* and *rifin*, which mediate antigenic variation and immune evasion (Smith et al., 1995; Scherf et al., 2008). This means that telomeres sit at the intersection of genome stability and host–parasite interactions, providing a unique therapeutic leverage point. Unlike generic targets that mainly affect parasite metabolism or growth, disrupting telomere maintenance could destabilize chromosome integrity and simultaneously impair immune evasion, striking at the parasite’s Achilles’ heel.

*Plasmodium* telomeres, characterized by unique 7-bp repeats and G-quadruplex (G4) structures, differ from human telomeres, offering potential for selective targeting (Edwards-Smallbone et al., 2022a). The subtelomeric regions of *Plasmodium* chromosomes contain most of the multicopy variant antigen-encoding genes that are key to the parasite’s virulence and play a major role in determining the severity of the disease (Smith et al., 1995; Su et al., 1995). Telomeres are distinctive not only because they address the end-replication problem of linear chromosomes, but also because of the lack of conservatively maintained terminal sequences across different chromosomes (Maestroni et al., 2017). Research on *Plasmodium* telomeres may uncover new drug targets, as single-celled eukaryotes require telomere maintenance for survival (Religa et al., 2014).

Although early efforts to target Plasmodium telomerase and telomeres faced challenges 18 related to specificity and efficacy, recent advancements in drug repurposing and structural biology have revitalized interest in this pathway (Mohanty et al., 2015a; Jha et al., 2022). Novel G-quadruplex stabilizers and small-molecule inhibitors originally developed for cancer are now being tested against parasitic systems, including Plasmodium (Calvo and Wasserman, 2016; Craven et al., 2023). Moreover, improved understanding of parasite-specific telomeric proteins such as PfTRZ and PfAP2Tel offers opportunities to selectively disrupt telomere maintenance without harming host cells (Sierra-Miranda et al., 2017a). These developments, coupled with the urgent need for new antimalarial strategies amid rising drug resistance, underscore the timely relevance of revisiting telomere biology as a therapeutic frontier in malaria research. In this review, we examine the unique characteristics of *Plasmodium* telomeres and telomerase, highlighting their roles in antigenic variation and immune evasion. Furthermore, we evaluate emerging telomere-targeting therapies, including G-quadruplex ligands and telomerase inhibitors, with a focus on their promising outcomes in preclinical studies.

## 2 Telomere structure, function, and unique challenges in plasmodium

Telomeres are nucleoprotein structures that cap chromosome ends, preventing degradation and preserving genome stability during replication (Blackburn, 1991; Lee and Pellegrini, 2023). In *Plasmodium falciparum*, telomeres consist of 7-bp repeats (GGGTTT/CA), forming guanine-rich overhangs that can fold into G-quadruplex (G4) structures (Vernick and McCutchan, 1988), in contrast to the human 6-bp repeat (TTAGGG) (Moyzis et al., 1988). These telomeric sequences are crucial for protecting chromosome ends and facilitating antigenic variation through subtelomeric virulence gene families such as *var* and *rifin* (Scherf et al., 2008).

Telomere shortening, a consequence of the end-replication problem, occurs with each replication cycle (Moyzis et al., 1988; Maestroni et al., 2017). In most eukaryotes, critically short telomeres trigger replicative senescence or apoptosis (Levy et al., 1992; Mason et al., 2024). However, *Plasmodium* circumvents this fate by maintaining continuous telomerase activity throughout its lifecycle, unlike humans, where telomerase is repressed in most somatic cells (Aldous et al., 1998; Fathi et al., 2019). This persistent activity ensures telomere stability during the parasite’s rapid intraerythrocytic proliferation and contributes to long-term survival.

**Figure 1:**
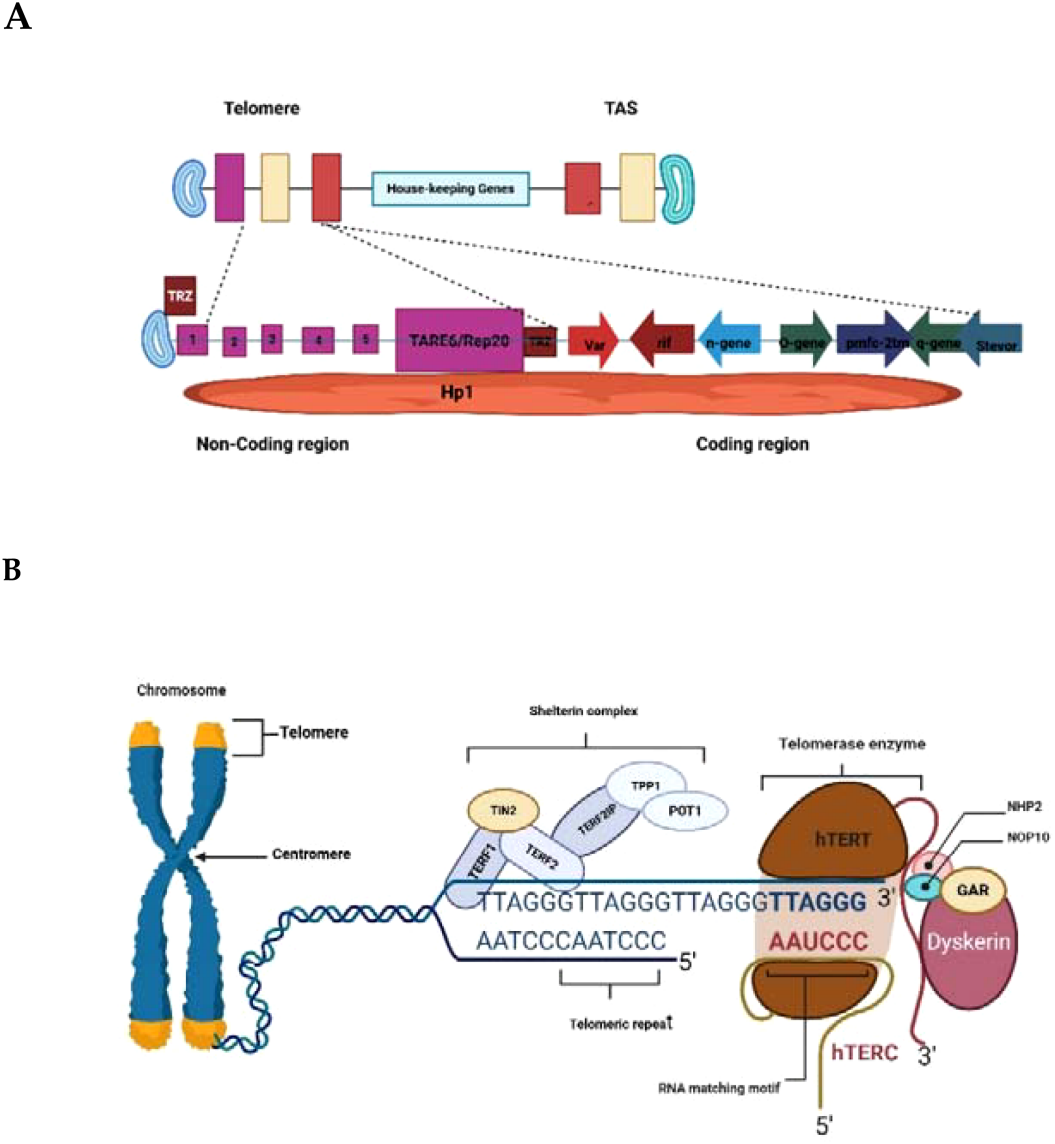
The Schematic structure of *P. falciparum* **(A)** and Human **(B)** telomere-telomerase Complexes. Created in BioRender. Wakai, T. (2025) *https://BioRender.com/izsde1g* ; *https://BioRender.com/hwsp15z* .

The architecture of *P. falciparum* chromosome ends further highlights its divergence from canonical eukaryotic models (**Figure 1**). Telomeric arrays are followed by 15–30 kb of telomere-associated sequences (TAS) organized into six TAREs (Sierra-Miranda et al., 2017a; Lee and Pellegrini, 2023) which house major virulence genes (*var, rif, stevor*) (Sriwilaijareon et al., 2002; Figueiredo et al., 2005). This positioning couples telomere dynamics directly to antigenic variation, enabling immune evasion. In addition, telomere-associated proteins such as PfTRZ, PfAP2Tel, and PfGBP2 stabilize G4 DNA, regulate chromatin states, and maintain genome integrity (Kirkman et al., 2014; Sierra-Miranda et al., 2017a; Edwards-Smallbone et al., 2022a). These functions are carried out by shelterin proteins in humans (Bertschi et al., 2017a; Button et al., 2022).

A unique challenge for *Plasmodium* telomere biology is the parasite’s highly A/T-biased genome. This bias reduces the frequency of guanine-rich sequences that are normally required for the formation of G-quadruplex (G4) DNA structures (Stewart et al., 2012). Despite this limitation, *Plasmodium* telomeres still form G4s that stabilize chromosome ends during replication. Interestingly, these parasite G4s differ in sequence context from canonical eukaryotic G4s, making them sensitive to small-molecule ligands that stabilize G4 folding. Such compounds could interfere with telomere maintenance and represent a novel class of antimalarial agents (Stewart et al., 2012; Jha et al., 2022). Despite this, *Plasmodium* telomeres remain sensitive to G4-stabilizing ligands (Calvo and Wasserman, 2016; Harris et al., 2018) highlighting their functional significance. Epigenetic regulators, such as the histone deacetylase PfSir2, further modulate subtelomeric chromatin and telomere length, linking telomere biology to virulence gene expression (De Cian et al., 2008; Duraisingh and Horn, 2016). These distinct adaptations of *Plasmodium* telomere biology diverge significantly from canonical eukaryotic models like *Homo sapiens*. In contrast to human telomeres, which primarily serve as passive protectors of chromosome ends and undergo progressive attrition, Plasmodium telomeres actively integrate genome stability with mechanisms of antigenic variation and survival. This evolutionary innovation not only sustains persistent infection but also highlights vulnerabilities that could be targeted for antimalarial drug development.

The structure of the Plasmodium falciparum chromosome end (**Figure 1A**) features the telomere repeat array (TRA), telomere-associated repeat elements (TARE 6/Rep20), and subtelomeric regions with virulence genes (var, rif, stevor). The non-coding region includes telomere repeat-binding proteins (e.g., PfTRZ) and histone proteins (e.g., Hp1), while the coding region contains housekeeping genes and other gene families (e.g., n-gene-o-gene, tmc-2m, q-gene). Telomere-Associated Sequences (TAS), organized into six TAREs with TARE 6/Rep20 being key, are vital for genome stability, antigenic variation, and immune evasion due to their role in housing virulence genes and regulating telomere maintenance. Human telomeres consist of repeated TTAGGG nucleotide sequences ending in a single-stranded, guanine-rich 3′ overhang. These ends are safeguarded by the shelterin complex, also known as the telosome (**Figure 1B**). It involves six core proteins. TERF1, TERF2, and POT1 directly interact with telomeric DNA, while TERF2IP, TIN2, and TPP1 function as bridging proteins within the complex. Telomerase, the enzyme responsible for extending telomeres, is composed primarily of hTERT (the catalytic subunit), hTERC (the RNA template), and DKC1. The hTERC subunit contains an H/ACA box domain that recruits DKC1 and its associated small nucleolar ribonucleoproteins, including NOP10, NHP2, and GAR1 (Bertschi et al., 2017a).

## 3 Telomerase and telomere maintenance in Plasmodium and other eukaryotes

Telomerase, comprising the catalytic subunit PfTERT and an RNA template, is responsible for maintaining telomere length in *Plasmodium (* Freitas-Junior et al., 2005). PfTERT, larger than its human counterpart due to unique sequence insertions, is essential for parasite replication, with knockout attempts in *P. berghei* failing to produce a viable clone (Religa et al., 2014). Unlike human telomerase, which is repressed in most somatic cells, Plasmodium telomerase is active throughout the parasite lifecycle, particularly during blood-stage proliferation (Aldous et al., 1998; Fathi et al., 2019). Telomere-associated proteins, including PfTRZ, PfAP2Tel, and PfGBP2, regulate telomere stability and gene expression (Bottius et al., 1998; Kirkman et al., 2014; Sierra-Miranda et al., 2017a). PfTRZ binds G4 structures, while PfAP2Tel uses a noncanonical AP2 domain to maintain chromosomal integrity (Sierra-Miranda et al., 2017a; Edwards-Smallbone et al., 2022b). These proteins, absent in humans, are potential therapeutic targets.

Generally, the telomeres of eukaryotic organisms are maintained predominantly by the ribonucleoprotein enzyme telomerase, which counteracts the progressive erosion of chromosome ends by synthesizing telomeric DNA repeats *de novo (* Cao, 2025). Telomerase comprises an RNA component that serves as the template and a catalytic protein subunit known as telomerase reverse transcriptase (TERT). The elongation of telomeres ensures that cells maintain sufficient chromosomal length to avoid premature replicative senescence or apoptosis, mechanisms intrinsic to cellular aging and tumor suppression (Lee and Pellegrini, 2023).

The shortening of telomeres beyond a critical threshold activates DNA damage signaling pathways, leading to irreversible cell cycle arrest or programmed cell death (Mason et al., 2024). Therefore, telomere homeostasis is crucial for cellular longevity and genome stability. Across diverse eukaryotes, including unicellular parasites such as *Plasmodium*, telomere maintenance mechanisms exhibit both conserved and unique features (Edwards-Smallbone et al., 2022b). For example, whereas most eukaryotes rely on canonical telomerase activity, some protozoan parasites utilize alternative telomere lengthening pathways or possess distinct telomere-associated proteins that modify telomere function (Sierra-Miranda et al., 2017a; Dey and Chakrabarti, 2018).

Understanding telomerase function and telomere dynamics in *Plasmodium* is foundational for deciphering mechanisms regulating parasite proliferation, genome integrity, and adaptation (Calvo and Wasserman, 2020). As telomere shortening and dysfunction are intimately linked with cellular outcomes in many organisms, the *Plasmodium* system offers an intriguing model to explore variations in telomere biology with implications for parasite survival and infectivity (Religa et al., 2014; Reed et al., 2021a).

Questions arise about how the parasite copes with replicating its telomeres; how it recalibrates the replicating telomeric DNA problem with the need to build the protective 3’ poly (T) cap; and how much of the promise of the rapidly expanding field of telomere biology can be harvested for therapy (Hu et al., 2022; Abraham, 2023). Curtailing the lifespan of the parasites or disrupting the protective telomeric cap they form could be a valuable adjunct to existing therapies. Many current antimalarials, such as artemisinin, act by generating reactive oxygen species (ROS) that damage vital parasite components (Vembar et al., 2016). While effective, this oxidative stress may also select for parasite subpopulations that adapt by enhancing telomere protection and stress resilience, potentially promoting resistance over time. Similar to findings in *Leishmania amazonensis*, where acute oxidative stress led to telomere shortening, DNA damage, and selective survival of fitter parasites with restored telomeres (Meshnick, 2002), it can be hypothesized that targeting telomere maintenance pathways in *Plasmodium* may disrupt parasite adaptation under drug-induced stress. Although telomere shortening alone may not result in immediate parasite death, impairing the parasite’s ability to recover telomere length could enhance the efficacy of existing therapies and reduce the likelihood of recrudescence (da Silva et al., 2017).

## 4 Components of telomere maintenance in *Plasmodium*

The catalytic heart of telomerase in *Plasmodium*, the telomerase reverse transcriptase (TERT) subunit, has been identified and characterized as unusually large relative to TERT components in other eukaryotes. This expanded size is attributed to unique sequence insertions and domain duplications that suggest species-specific adaptations (Sriwilaijareon et al., 2002). PfTERT is expressed during key intraerythrocytic stages and localizes at discrete nuclear compartments associated with the nucleolus rather than the canonical telomeric clusters seen in model organisms, a phenomenon indicative of divergent telomerase organization in *Plasmodium (* Fathi et al., 2019; Edwards-Smallbone et al., 2022b).

Genetic studies have demonstrated that TERT is essential for parasite survival, as attempts to knock out the TERT gene in rodent malaria species (*P. berghei*) resulted in failure to obtain viable clones (Religa et al., 2014). This underpins the indispensable role of telomerase-catalyzed telomere elongation during parasite replication, reinforcing its candidacy as a critical target for antimalarial strategies. The presence of functional telomerase activity compensates for telomere shortening, ensuring sustained proliferative capacity vital for the parasite’s life cycle continuity. Further molecular characterization revealed that telomerase RNA components complement TERT function to maintain telomeric DNA at a relatively constant length during blood-stage proliferation (Religa et al., 2014; de Oliveira et al., 2024). The discovery and delineation of the *Plasmodium* TERT thus provide important insights into telomere biology.

Beyond telomerase itself, several telomere-associated binding proteins have been identified in *Plasmodium* that interact directly with telomeric DNA structures, supporting telomere maintenance and genome integrity. Among these, PfGBP2 (G-strand Binding Protein 2) has been characterized as a multifunctional DNA/RNA-binding protein that specifically engages with G-quadruplex DNA and RNA structures present at telomeres (Bottius et al., 1998). Its interaction with G-rich quadruplexes and RNA, both *in vitro* and *in vivo*, suggests a role in stabilizing telomeric structures and possibly regulating gene expression linked to telomere biology (Edwards-Smallbone et al., 2022a; Edwards-Smallbone et al., 2022c). Other telomere-associated proteins include PfAP2Tel and PfTRF, which belong to the ApiAP2 transcription factor family and telomere-repeat binding factors, respectively (Sierra-Miranda et al., 2017a). PfAP2Tel has been shown to specifically bind telomeric DNA repeats via a noncanonical DNA-binding AP2 domain and is involved in the maintenance of chromosomal integrity (Sierra-Miranda et al., 2017a). PfTRF, distinct from classic telomere repeat binding factors in other eukaryotes, binds telomeric repeats using a C-terminal C2H2-type zinc finger domain, and is essential for mitotic progression and telomere length regulation (Kirkman et al., 2014; Gurung et al., 2021). These proteins collectively form a unique molecular complex divergent from other eukaryotic systems yet adapted to fulfill the indispensable requirement of chromosome end protection in *Plasmodium (* Fathi et al., 2019). Their distinct binding specificities and modes of action reflect evolutionary divergence and specialization, emphasizing both the complexity and uniqueness of telomere maintenance in the parasite.

## 5 Regulatory factors affecting *Plasmodium* telomere maintenance

Regulation of telomere biology in *Plasmodium* extends into the epigenetic domain, with factors such as PfSir2 (a histone deacetylase) and PfHP1 (heterochromatin protein 1) playing pivotal roles in subtelomeric gene silencing and telomere length control. PfSir2 contributes to the repression of variant antigen gene families and modulates telomere length, functioning as a gatekeeper of chromatin state at chromosome ends (da Silva et al., 2017).

Molecular chaperones such as PfHsp90 have been implicated in regulating the stability and activity of PfSir2, where direct interaction maintains PfSir2 function. Chemical inhibition of PfHsp90 leads to depletion of PfSir2 protein and consequent loss of its histone deacetylase activity, resulting in the derepression of ribosomal genes and possibly other subtelomeric loci (Zheng et al., 2019). This regulatory axis exemplifies the intricate control over telomere-associated chromatin-modifying enzymes.

Physiologically relevant stimuli, such as febrile temperature, typical of malaria infection, induce transcriptional downregulation of PfSIR2A and PfSIR2B mediated by PfHsp90, leading to altered telomeric chromatin landscape and gene expression changes (Tabassum et al., 2022). This modulation of telomere maintenance machinery during infection adds a dynamic layer to parasite adaptability and may represent a stress-responsive epigenetic mechanism facilitating parasite survival under host immune pressure (De Cian et al., 2008).

## 6 Comparison between *Plasmodium* and human telomere–telomerase systems

While human telomerase is typically active in embryonic cells, germline cells, and certain stem cells, it is usually repressed in most somatic cells (Tabassum et al., 2021). Aberrant activation or alterations in telomere maintenance can lead to complications such as cancer, where unchecked telomere elongation enables unlimited cell proliferation (Akincilar et al., 2016; Panasiak et al., 2023). In *P. falciparum*, telomerase activity has been detected in semi-purified nuclear extracts from the blood stages (Aldous et al., 1998; Guterres and Villanueva, 2020). This enzyme maintains telomere length and may also repair broken chromosome ends (Sriwilaijareon et al., 2002; Freitas-Junior et al., 2005). Telomerase is critical for ensuring proper telomere maintenance during the parasite’s rapid replication, especially in the bloodstream (Abraham, 2023). The gene encoding the telomerase reverse transcriptase (TERT) protein in *Plasmodium* has unique features that define its telomere-telomerase dynamics (Sriwilaijareon et al., 2002). The subtelomeric regions of malaria parasites contain virulence genes and undergo telomere healing in response to DNA damage (Raj et al., 2003). Like cancer cells, constant telomerase activity is essential for the survival of ancient eukaryotic pathogens such as *Plasmodium*, Trypanosoma, and Leishmania (Cao, 2025). The biochemical functions of telomerase in these organisms are not entirely understood, but their similarity could offer new therapeutic options. Notably, in parasites like *Trypanosoma*, changes in telomere length can impact antigenicity, influencing disease pathology (Reed et al., 2021b). Moreover, unlike human somatic cells, where telomerase is typically inactive, *Plasmodium* maintains active telomerase throughout its lifecycle, particularly during blood-stage proliferation (Sriwilaijareon et al., 2002; Cao, 2025).

In humans, telomerase is generally repressed, leading to gradual telomere shortening. *Plasmodium* maintains stable telomere length through tight regulation (World Health Organization, 2023). As would be expected, their telomeres grow and recombine rapidly, unlike the more stable dynamics seen in human telomeres (Raj et al., 2003; Freitas-Junior et al., 2005; Li, 2023). In humans, telomerase consists of RNA and protein components. While telomerase is typically repressed in most somatic cells, it remains active in certain cell populations such as embryonic stem cells and the majority of cancer cells, where it facilitates continuous proliferation by counteracting telomere shortening (Simantov et al., 2022). In contrast, most normal somatic cells undergo telomere attrition with each cell division, ultimately leading to replicative senescence (Mason et al., 2024). The activity and regulation of telomerase are heavily influenced by a group of telomere-associated proteins known collectively as the shelterin complex (Li, 2015; Shay and Wright, 2019).

*P. falciparum* employs two mechanisms—homologous recombination and *de novo* telomere addition—to repair double-strand breaks in subtelomeric regions, allowing a balance between genome stability and antigenic diversity (Raj et al., 2003; Diotti and Loayza, 2011). In this parasite, telomerase interacts with a distinct set of telomere-associated proteins, differing from well-characterized complexes in humans (Bottius et al., 1998; Kirkman et al., 2014). These structural and compositional differences may render parasite telomeres more susceptible to targeted disruption, offering a potential avenue for selective antimalarial intervention (Vernick and McCutchan, 1988; Calhoun et al., 2017). Some special proteins, such as *Plasmodium* falciparum Telomere Repeat-binding Zinc finger protein (PfTRZ) and Apetala 2 Telomere protein (PfAP2Tel), are unique telomere-associated proteins in *Plasmodium*. PfTRZ binds telomeric DNA and regulates the expression of 5S rRNA, while PfAP2Tel is involved in telomere maintenance (Sierra-Miranda et al., 2017b; Yasir et al., 2025). Targeting these proteins could impair *Plasmodium* telomere maintenance without affecting human telomeric structures, offering. The unique N-terminal extension of PfTERT and the absence of a conserved template region in *ter 1* RNA contrast sharply with human telomerase components (Bonnell et al., 2025; Davis and Chakrabarti, 2022). Comparative biochemistry of telomerase systems in humans and *Plasmodium* will provide clues on novel targets against the parasite, as shown in **Table 1**.

**Table 1:** Comparison between *Plasmodium* and human telomere–telomerase systems.

Feature	Plasmodium telomerase system	Human telomerase system	References
Telomere repeat sequence	**GGGTT(T/C)A**	TTAGGG	(Vernick and McCutchan, 1988; Wyatt et al., 2010)
Telomerase RNA template	***ter1* (lacks conserved template region)**	hTERC (conserved template)	(Greider and Blackburn, 1989; Edwards-Smallbone et al., 2022b)
Telomerase reverse transcriptase	**PfTERT(large, unique N-terminal extension)**	hTERT (canonical structure)	(Sriwilaijareon et al., 2002; Calhoun et al., 2017)
Accessory proteins	**PfTRZ, PfAP2Tel, PfGBP2, PfTRF**	Shelterin complex (TRF2, TPP1, TIN2, POT1)	(Kirkman et al., 2014; Bertschi et al., 2017a)
Telomerase activity regulation	Continuous throughout the parasite lifecycle	Repressed in most somatic cells; regulated by the cell cycle	(Simantov et al., 2022)

Bold values indicate features that are unique to Plasmodium and differ significantly from the corresponding human telomere–telomerase system.

*Plasmodium* telomeres grow and recombine rapidly, a stark contrast to the more stable dynamics seen in human telomeres (Raj et al., 2003). This parasite relies on both telomerase and recombination for telomere maintenance, differing from the primarily telomerase-driven process in humans (Diotti and Loayza, 2011; Cao, 2025). Moreover, *Plasmodium*’s telomerase system, regulated by telomere-associated proteins and the cell cycle, has evolved uniquely to meet the demands of the parasite’s lifecycle and may also aid in evading the host’s immune system (Belachew, 2018). Several telomere-associated proteins have been identified in *P. falciparum* strains. Noteworthy proteins include PfTRZ, which binds telomeric DNA and regulates 5S rRNA expression (Kirkman et al., 2014); PfAP2Tel, which has an atypical AP2 domain and binds telomeric DNA (Sierra-Miranda et al., 2017a) and PfGBP2, which interacts with G-quadruplex structures and G-rich RNAs. Among these, only PfTRZ has been confirmed to play an essential role in telomere maintenance (Kirkman et al., 2014).

**Table 2** shows a comparison of telomere-associated protein complexes in Plasmodium and related parasites. This helps to understand their roles and how they could be targeted for treatment, while also considering possible effects on the human host or other parasites

**Table 2:** Comparison of telomere-associated protein complexes in *Plasmodium* and related parasites.

Component	Description	Role in Plasmodium	Therapeutic potential	References
Telomeric DNA (GGGTT(T/C)A)	Unique repeat sequence with base J modification	Protects chromosome ends, involved in gene silencing	Targeting base J synthesis or recognition could disrupt telomere stability	(Harris et al., 2018)
PfSir2a/b	NAD+-dependent histone deacetylases	Regulate telomeric/subtelomeric gene silencing (e.g., var genes), critical for antigenic variation	Inhibitors could disrupt immune evasion, but risk of cross-reactivity with host HDACs	(Duraisingh et al., 2005; Duraisingh and Horn, 2016),
Telomerase (PfTERT, RNA template)	Ribonucleoprotein reverse transcriptase	Maintains telomere length during rapid proliferation (schizogony, sporogony)	Telomerase inhibitors (e.g., small molecules, antisense oligonucleotides) could induce replicative senescence	(Religa et al., 2014)
Telomere-binding proteins	Functional analogs to shelterin (not fully characterized)	Protect telomeres, regulate replication	Parasite-specific domains could be targeted to avoid host toxicity	(Kirkman et al., 2014; Edwards-Smallbone et al., 2022a),

## 7 G-Quadruplexes in plasmodium telomeres

Despite the A/T-rich *Plasmodium* genome, G4 structures form in telomeric and subtelomeric regions, stabilizing telomeres and modulating virulence gene expression (Anas et al., 2017; Harris et al., 2018). Targeting G4s offers a novel strategy, though specificity remains a challenge due to potential host G4 interactions. Certain DNA or RNA sequences rich in guanine can fold into a unique four-stranded shape known as a G-quadruplex. When these structures form within gene promoter regions, they have the potential to suppress gene activity (Smargiasso et al., 2009). G-quadruplexes (G4s) are noncanonical four-stranded nucleic acid secondary structures formed by stacked guanine tetrads stabilized through Hoogsteen hydrogen bonding (Stewart et al., 2012). In *Plasmodium* falciparum, despite an extremely A/T-rich genome which limits the prevalence of guanine-rich sequences, G4 motifs have been identified primarily in telomeric and subtelomeric regions (Smargiasso et al., 2009). Their presence was confirmed not only via in silico predictions but also experimentally through biophysical and biochemical assays demonstrating that these G-rich repeats can fold into stable G4 structures *in vitro* and presumably *in vivo (* Stewart et al., 2012). In terms of functionality, the G4 structures contribute to telomere maintenance by affecting telomerase activity, regulating the accessibility of telomere-associated proteins, and playing roles in genome stability in humans (Lombardi, 2011). They also influence recombination events, which are critical in generating diversity in subtelomeric virulence gene families (Smargiasso et al., 2009; Stanton et al., 2016). The strategic distribution of G4 motifs near variant antigen genes suggests their involvement in facilitating gene rearrangements essential for antigenic variation (Witmer et al., 2012; Bertschi, 2015). The identification of G4 motifs and their stabilizing proteins in *Plasmodium* underscores their biological significance in telomere dynamics and genome architecture, promising avenues for antimalarial drug development targeting these unique DNA structures (Belmonte-Reche et al., 2018).

**Figure 2:**
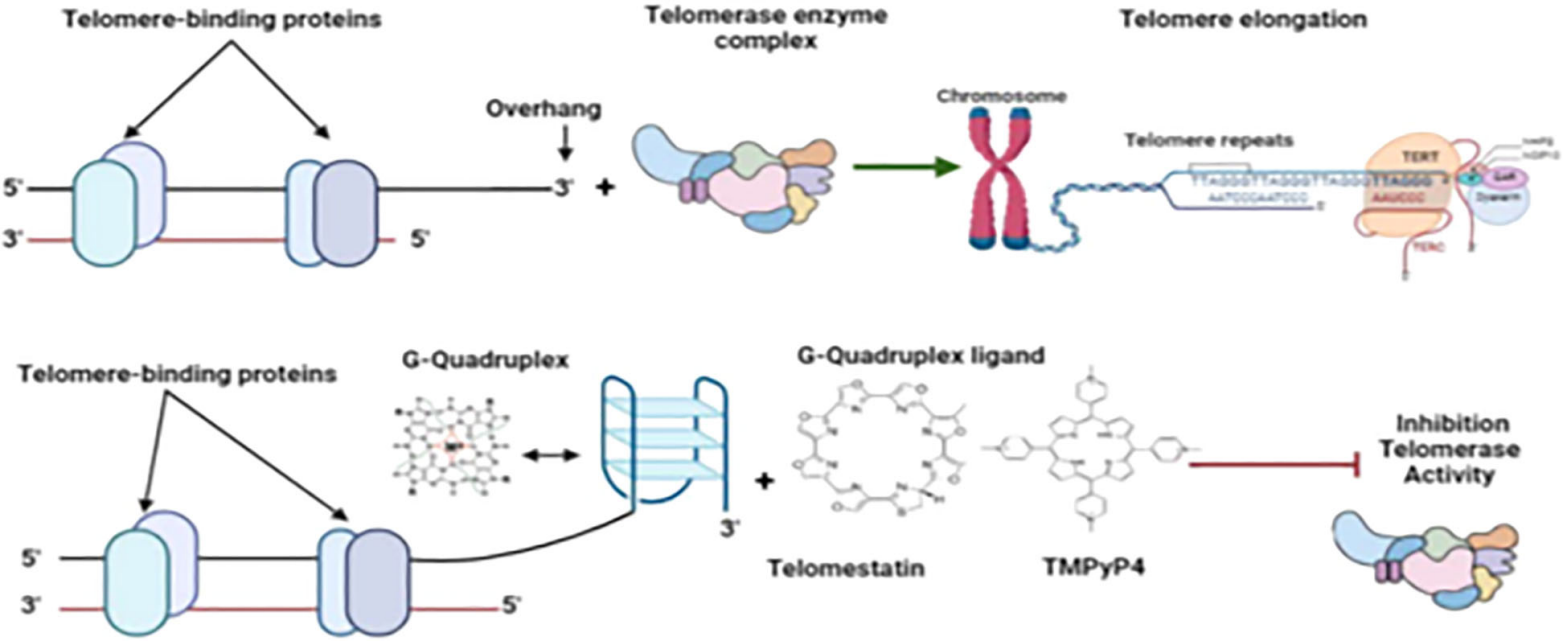
Inhibition of telomerase by G-quadruplex ligands. Figure by T. N. Wakai, designed in BioRender.com, adopted from Calvo and Co (Dey and Chakrabarti, 2018). Reprinted from *Molecular and Biochemical Parasitology*, Vol 207, Pages 33-38, with permission from Elsevier.

Exploiting the unique presence and functional importance of G-quadruplexes in *Plasmodium* telomeres offers promising therapeutic avenues. Certain G4-stabilizing compounds, such as quarfloxin, initially developed for anticancer indications, have demonstrated potent antiplasmodial activity, including rapid parasiticidal effects at multiple life cycle stages (Smargiasso et al., 2009; Stewart et al., 2012). These agents stabilize G4 structures, which may disrupt processes such as telomere maintenance or transcriptional regulation that are critical for parasite survival. Interestingly, *P. falciparum* shows sensitivity to these compounds despite having relatively few predicted G4 motifs, underscoring the potential functional significance of these rare structures (Religa et al., 2014; Belmonte-Reche et al., 2018). Achieving specificity and reducing off-target effects in the host is worth investigating. Investigation into parasite-specific G4-binding proteins and the precise mechanisms of G4-mediated regulation will facilitate the design of selective G4-targeted antimalarials. The convergence of telomere biology and pharmacology in this domain underscores the potential for innovative therapeutic strategies (Anas et al., 2017), as highlighted in **Figure 2**.

## 8 Current and potential telomerase inhibitors

Recent advances in our understanding of the structural differences between *Plasmodium* and human telomerase components offer a pathway to designing more selective inhibitors (Bonnell et al., 2025; Edwards-Smallbone et al., 2022b; Udroiu et al., 2022). The differences between *Plasmodium* and human telomerase systems make *Plasmodium* telomerase an attractive target for developing new antimalarial drugs (Bottius et al., 1998; Belmonte-Reche et al., 2018; Dey and Chakrabarti, 2018; Morozova et al., 2020). This system is essential for maintaining chromosome stability during replication, which permits continuous replication and induces damage to the host’s red blood cells (Cao, 2025) (Bertschi, 2015). Targeting this system emerges as a promising focus for both diagnostic and therapeutic applications against *Plasmodium* parasites, with the potential to disrupt replication and control spread (Li, 2012; Ali and Walter, 2023.

While telomerase research has largely centered on cancer, exploring telomeres and telomerase-targeting strategies is also vital in infectious diseases, particularly malaria (Ilmonen et al., 2008; Giraudeau et al., 2019; Morozova et al., 2020). Several telomerase inhibitors identified in cancer research, including nucleoside analogs and small molecules targeting the reverse transcriptase domain, have potential for repurposing as antimalarial agents (Mohanty et al., 2015a; Sierra-Miranda et al., 2017c). These compounds can inhibit telomerase activity by interfering with its catalytic function or disrupting its assembly (Fathi et al., 2019). Studies focusing on *Plasmodium* have highlighted compounds capable of perturbing telomere synthesis *in vitro*, suggesting feasibility for selective inhibition (Dey and Chakrabarti, 2018). However, developing inhibitors that selectively target *Plasmodium* telomerase without affecting the human enzyme is challenging because their core domains are conserved, even though the parasite’s telomerase has some unique features. Research has suggested that specific inhibitors can disrupt telomerase activity in *Plasmodium*, potentially reducing parasite viability. For instance, in silico modeling has identified small-molecule nucleoside analogs that selectively bind and inhibit *P. falciparum* telomerase reverse transcriptase without targeting the human enzyme (Mohanty et al., 2015a). Complementing this, *in vivo* studies have demonstrated that certain telomerase inhibitors can impair parasite growth, supporting the potential for telomerase-targeted antimalarial strategies (Sierra-Miranda et al., 2017c) (e. Moreover, the properties of the *Plasmodium* telomerase complex, including its interactions with unique telomere-associated proteins, provide additional potential targets for drug design. For example, the PfTRZ and PfAP2Tel proteins are integral to the parasite’s telomere maintenance and may serve as potential targets for novel therapeutics (Bottius et al., 1998; Mohanty et al., 2015b). By inhibiting telomerase, it would be possible to induce telomere shortening, genomic instability, and ultimately parasite death (Abraham, 2023). Advancing this pharmacological space involves structural characterization of the parasite telomerase complex, identification of accessory proteins, and screening for compounds that exploit differences between host and parasite enzymes (Bottius et al., 1998). The pursuit of telomerase inhibitors in malaria therapy, therefore, is promising but demands overcoming challenges related to selectivity and efficacy (Guillon et al., 2022). The compounds reported to target, or with potential to target, *Plasmodium* telomere maintenance systems through G-quadruplex stabilization, telomerase inhibition, or related mechanisms are summarized in **Table 3**.

**Table 3:** Reported and potential compounds targeting telomere maintenance in plasmodium.

Compound	Description	Mechanism of action	Therapeutic potential	References
TMPyP4	5,10,15,20-Tetra(N-methyl-4-pyridyl)porphyrin	Stabilizes G-quadruplexes, inhibits telomerase activity	Reduces *P. falciparum* proliferation	(Dey and Chakrabarti, 2018)
Telomestatin	Macrocyclic natural product	Stabilizes G-quadruplexes, inhibits telomerase	Strongly impairs parasite growth	(Calvo and Wasserman, 2016)
Bis-pyrrolo[1,2-a]quinoxalines (1n, 1p)	Polycyclic heterocyclic derivatives	Stabilizes *Plasmodium* telomeric G-quadruplexes	Antimalarial activity via telomere interference	(Chakravarti et al., 2021)
AZT-TP	Azidothymidine triphosphate	Nucleoside analog inhibiting PfTERT	Blocks telomere elongation	(Guterres and Villanueva, 2020)
7-deaza-dGTP	7-deaza-2’-deoxyguanosine-5’-triphosphate (Guanine analog)	Competes for incorporation, inhibits PfTERT	Prevents telomere extension	(Guterres and Villanueva, 2020)
Vidarabine	Adenine arabinoside	Predicted to bind the PfTERT active site	Blocks reverse transcription	(Witmer et al., 2012)
Berberine	Isoquinoline alkaloid	Inhibits telomerase activity dose-dependently	Impairs intraerythrocytic growth	(Fathi et al., 2019)
ddGTP	Dideoxyguanosine triphosphate	Chain terminator of telomerase	Inhibits telomere extension	(Freitas-Junior et al., 2005)
Floxuridine, Gemcitabine, Stavudine	Nucleoside analogs	High affinity to PfTERT (docking studies)	Inhibit the reverse transcriptase domain	(Guillon et al., 2022)
17-AAG (Tanespimycin)	17-allylamino-17-demethoxygeldanamycin	Depletes PfSir2, alters histone acetylation	Shields telomerase access to telomeres	(Belmonte-Reche et al., 2022)
Quarfloxin	6-fluoro-3-[(2S,4S)-4-methyl-2-piperidinyl]-1H-pyrido[4,3-b]indole-1,7(8H)-dione	Binds Pf-encoded G4 sequences	Potent against blood-stage malaria in the murine model	(Belmonte-Reche et al., 2018)
CX-5461	1-[2 (dimethylamino)ethyl]-2-(hydroxyimino)-6-methyl-4-oxo-1,4-dihydroquinoline-3-carbonitrile	Stabilizes G-quadruplexes, inhibits RNA Pol I–mediated rRNA transcription	Disrupts parasite genome stability	(Calvo and Wasserman, 2016)

## 9 Risks of off-target effects and strategies to minimize toxicity for future drug development

Although telomere–telomerase systems of *P. falciparum* are attractive targets for therapy, there are important risks associated with their evolutionary conservation in humans. Human telomerase enzyme, while repressed in most somatic cells, is essential in stem cells, germline tissues, and hematopoietic progenitors (Simantov et al., 2022; Mason et al., 2024). Broad inhibition may impair tissue renewal, accelerate senescence, or trigger cytotoxicity in these proliferative compartments. Similarly, G-quadruplex (G4) DNA structures, which also exist in human telomeres and gene promoters, are vital for genome stability and regulation of transcription (Stewart et al., 2012; Belmonte-Reche et al., 2022). Small-molecule G4 stabilizers developed for *Plasmodium* may therefore risk off-target stabilization of host G4s, with potential genotoxic effects.

Resistance development can potentially arise from mutations in telomerase components or alternative telomere lengthening pathways. Hence, a comprehensive understanding of telomere biology and redundancy in maintenance mechanisms is essential to design combination therapies that reduce resistance risk. Moreover, integrating telomerase-targeting compounds with existing antimalarial regimens could enhance therapeutic efficacy, potentially eradicating parasite populations persisting through resistance or dormancy (Morozova et al., 2020). This holistic approach situates telomere maintenance inhibitors within the broader landscape of malaria control. Drugs specifically designed to target telomeres and telomerase have shown promise in cancer therapy (Akincilar et al., 2016; Giraudeau et al., 2019) but their role in malarial parasites could be repurposed (Calvo and Wasserman, 2016; Mohanty et al., 2015a).

Several strategies can minimize these risks. First, selective inhibitor design should exploit parasite-specific features such as the unusually large PfTERT catalytic subunit with its N-terminal extension and unique sequence insertions (Sriwilaijareon et al., 2002; Religa et al., 2014). Targeting these distinct domains, absent in humans, can reduce cross-reactivity. Second, focusing on parasite-exclusive telomere-associated proteins such as PfTRZ, PfAP2Tel, and PfGBP2—proteins with no homologues in human telomere biology—offers additional avenues for specificity (Sierra-Miranda et al., 2017a; Edwards-Smallbone et al., 2022a). The possibility of designing combination therapy with existing antimalarials could allow dose reduction, thereby mitigating toxicity while delaying resistance (Mohanty et al., 2015a; Menard and Dondorp, 2017). Finally, innovative delivery approaches such as nanoparticle-based formulations or ligand-guided carriers may enhance parasite-specific accumulation and limit systemic exposure (Craven et al., 2023) (Calvo and Wasserman, 2016).

**Figure 3:**
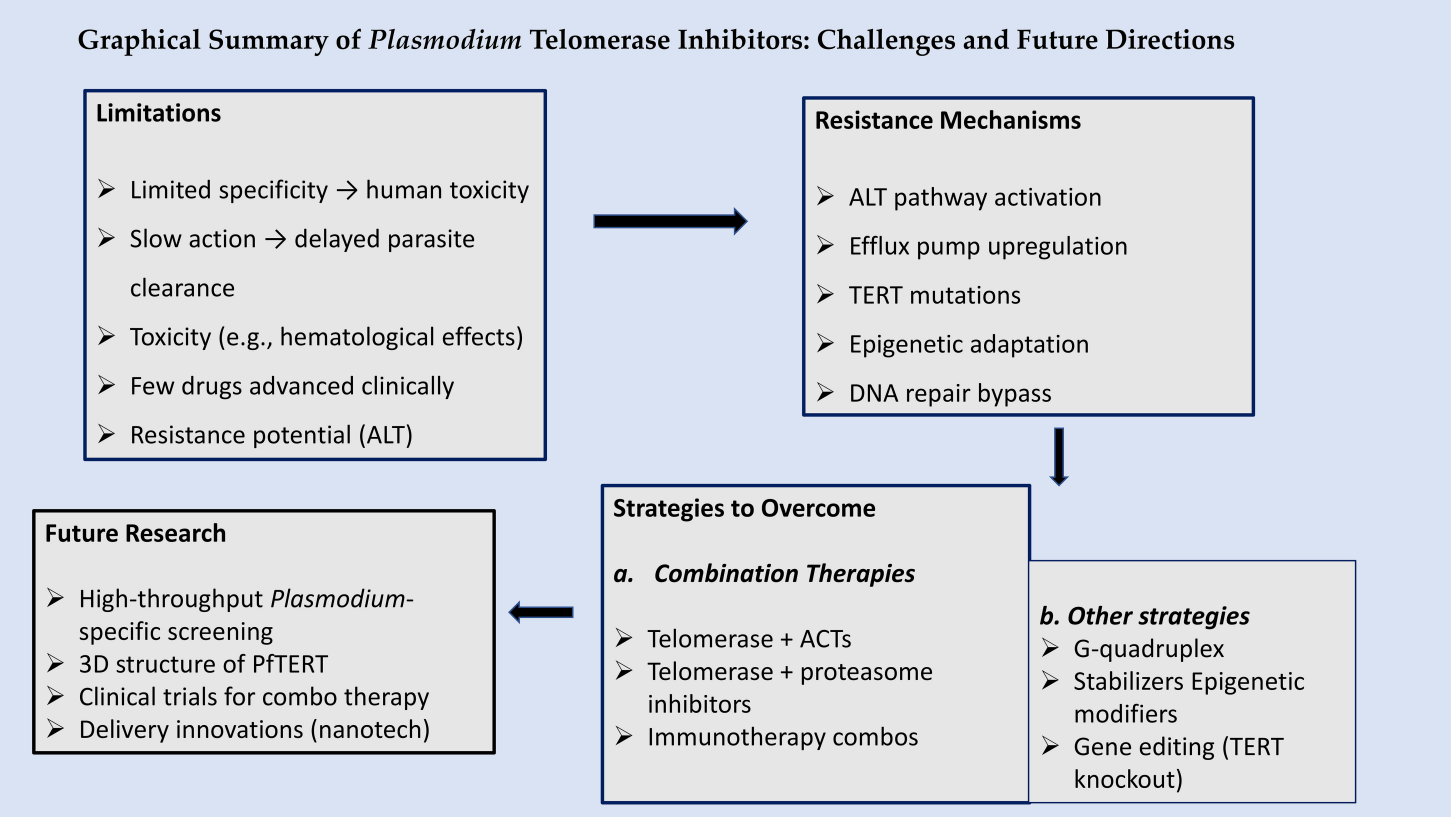
Graphical summary of therapeutic barriers and opportunities in *Plasmodium* telomerase inhibition. *Crafted by TN Wakai, via Canva design tool (* *https://www.canva.com/* *)*.

**Figure 3** summarizes the key challenges and future directions in targeting *Plasmodium* telomerase. Limitations include conserved telomerase structure between parasite and host (Cao, 2025), toxicity seen with inhibitors like imetelstat (Bertschi et al., 2017b; Lennox et al., 2024) and reliance on recombination-based telomere maintenance (Diotti and Loayza, 2011). Resistance may emerge via efflux pumps (*PfMDR1*), ALT activation, or epigenetic adaptation (Tabassum et al., 2022). Overcoming these barriers will require parasite-specific drug design targeting PfTERT’s unique domains and telomere-binding proteins such as PfTRZ and PfAP2Tel (Sierra-Miranda et al., 2017a), along with combination therapy and innovative delivery systems like CRISPR vectors and nanoparticles (Lennox et al., 2024). Future research should prioritize high-throughput screening, structural studies, and resistance monitoring (Bonnell et al., 2025; Calvo and Wasserman, 2020).

## 10 Conclusion

Telomere maintenance is a critical Achilles’ heel for *Plasmodium* parasites, and it offers a compelling target for novel malaria control strategies. The parasite’s dependence on continuous telomerase activity for survival and its unique telomere-associated proteins, such as PfTRZ and PfAP2Tel, reveal intervention points that differ significantly from those in humans and could enable selective therapeutic approaches. Furthermore, the presence of G-quadruplex structures in this parasite genome’s telomeric and subtelomeric regions presents an additional opportunity to disrupt telomere stability and parasite survival. While these targets are promising, challenges persist, including the risk of off-target effects due to telomerase conservation across species and the parasite’s capacity to switch between telomerase-dependent and recombination-based telomere maintenance. Addressing these obstacles requires a combination of carefully designed inhibitors and a deeper understanding of parasite-specific telomere biology. The *Plasmodium* telomere–telomerase system highlights a key vulnerability in the parasite’s genome maintenance machinery, which biologists could exploit to develop next-generation antimalarial therapies.
